# Youth preferences for healthcare providers and healthcare interactions: a qualitative study

**DOI:** 10.1186/s12875-024-02300-z

**Published:** 2024-02-21

**Authors:** Marika Waselewski, Xochitl Amaro, Ryan Huerto, Jessica Berger, Marcus Spinelli da Silva, Kate Siroky, Anthony Torres, Tammy Chang

**Affiliations:** 1 Department of Family Medicine, University of Michigan, 2800 Plymouth Road, Building 14 G128, Ann Arbor, MI 48109 USA; 2 Department of Adult and Family Medicine, Kaiser Permanente Northern California, Oakland, CA USA; 3 School of Medicine, University of Michigan, Ann Arbor, MI USA; 4 Institute for Healthcare Policy and Innovation, University of Michigan, Ann Arbor, MI USA; 5 University of Michigan, Ann Arbor, MI USA

**Keywords:** Adolescent healthcare, Patient-physician relationships, Concordance

## Abstract

**Background:**

Patient-physician relationships in healthcare can influence healthcare provision, patient engagement, and health outcomes. Little is known about youth preferences on types and characteristics of their healthcare providers. The aim of this study was to assess youth perspectives on preferences for and interactions with their healthcare providers.

**Methods:**

We posed 5 open-ended questions to 1,163 MyVoice participants, a nationwide text message cohort of United States youth aged 14–24, on April 10, 2020 related to youth preferences for healthcare providers. Content analysis was used to develop a codebook. Responses were independently coded by two reviewers with discrepancies discussed to reach consensus. Descriptive statistics were calculated for demographics and frequency of codes.

**Results:**

944 (81%) participants responded to at least one question. Respondents had a mean age of 18.9 years (SD: 2.8) and were a majority female (53.6%) and White (56.3%). Youth reported “kindness” or other personality traits (31%) and education (30%) as important in choosing their doctor. Patient-physician concordance was not important to many youths (44%) and among those who reported concordance as important (55%), having the same gender was the most noted (68%). Youth suggested respect, open conversation, and addressing issues directly to help alleviate uncomfortable situations, though some would simply switch providers.

**Conclusion:**

Personality and empathy are important provider characteristics valued by youth. Female respondents preferred gender concordant providers, particularly for sexual health-related issues, and non-white respondents were more likely to prefer racial concordance. Strengthening professional and interpersonal skills among youth-serving providers may improve healthcare engagement and satisfaction among youth.

## Introduction

Patient-physician relationships in healthcare can influence healthcare provision, patient engagement, and health outcomes [1–3]. Trust, knowledge, regard, and loyalty have previously been described as the 4 components that frame the patient-physician relationship and its impact on health outcomes [4]. Patient factors (e.g., prognosis, new patient, health literacy), provider factors (e.g., career stage, burnout), mismatch factors (e.g., language, culture), and systemic factors (e.g., time, space) can all interfere with this relationship [4]. Additionally, preconceived expectations and biases related to sociodemographic characteristics, like race or social class, can also influence patient-physician relationships and health outcomes among patients [5, 6]. Sociodemographic characteristics of patients including age, gender, race, sexual orientation, and education are also associated with disparities in the provision of healthcare and health outcomes [5, 7].

Patient-physician social concordance, or similarity of sociodemographic characteristics such as race/ethnicity, gender, sexual orientation, disability status, culture, or language, between patients and providers [5], has been suggested as an important factor in patient-physician relationships and the quality of healthcare delivery and patient outcomes [5, 6, 8]. Patients may experience improved adherence, more timely healthcare delivery, greater shared decision-making, better communication, and higher satisfaction when seen by racially concordant providers [5, 9–17]. Patient-physician concordance has additionally been linked to higher patient experience ratings among parents of minority youth [18]. However, these studies have only assessed adult perspectives of their own care or the care of their children and these results are mixed [19, 20]. Less is known regarding the role of patient-physician concordance, and its impact on health outcomes and healthcare interactions among adolescents and young adults [21].

Patient-physician relationships are particularly important among youth who may be entering the healthcare system alone for the first time and have unique concerns of confidentiality, embarrassment, or judgement related to health behaviors [22]. Youth often feel unprepared to independently navigate the healthcare system and young adults are less likely to access healthcare services compared to teens and older counterparts [22, 23]. Greater understanding of youth perspectives on patient-physician relationships can inform programs and policies that improve youth engagement in healthcare and health outcomes. Therefore, the aim of this study was to understand youth perspectives of their relationships with their healthcare providers and the importance of sociodemographic similarities and differences as reported by youth.

## Methods

Data was collected via MyVoice, a nationwide text message poll of youth aged 14–24 years [24]. Targeted recruitment was performed through social media advertisements based on national benchmarks for age, gender, race, and census region from weighted samples of the American Community Survey. Demographic data, as well as contact information, were collected in an online survey upon consent for the study, including age, sex, race, ethnicity, level of education, qualification of free or reduced-price school lunch, and census region.

Five open-ended questions were sent via text message to all active MyVoice participants on April 10, 2020 and participants had a week to respond. The questions were created by a team of experts in youth-centered text message survey design, including youth. The 5 questions posed to youth were (1) This week we want to know what you would look for when choosing a doctor. What characteristics are important to you, if any? Why?; (2) Is it important that you have similar characteristics to your doctor (age, gender, race/ethnicity, disability, background, personality, etc.)? If so, what characteristics?; (3) When would being similar to your doctor be important? Specifically, for what type of healthcare visits?; (4) How would being similar to your doctor impact what you say or do during your visit?; (5) If you were in a situation where you felt uncomfortable with your doctor, what could they do to make you feel more comfortable?

Prior to analysis, survey responses are merged with respondent demographic characteristics and deidentified. Two researchers reviewed all open-ended qualitative responses to iteratively develop a codebook of major concepts reported by respondents using content analysis. These codes were then independently applied to the entire dataset by two investigators and organized into major themes. Any discrepancies in coding were discussed to reach a consensus to ensure validity in final categorizations of codes. Demographics and frequency of codes were summarized using descriptive statistics (SAS 9.4). Code frequencies of respondents’ perspectives on the importance of concordance were also compared using chi-square testing.

Investigators responsible for coding and describing major themes included two family medicine physicians, four undergraduate research assistants, one medical student, and a research project manager. All investigators have no relationship to participants and no reported personal attributes or experiences that may influence the research beyond their own experiences with healthcare.

This study was approved by the University of Michigan Institutional Review Board (HUM00119982) including a waiver of parental consent for minor participants. Online consent, or assent for minors, was obtained from all participants. American Association for Public Opinion Research (AAPOR) and the Standards for Reporting Qualitative Research (SRQR) guidelines for survey research are followed in reporting [25–27].

## Results

A total of 944 participants responded to at least one question (response rate: 81%, 944/1,163). Respondents (Table 1) had a mean age of 19 years (SD: 2.8), were a majority female (54%), white (56%), and had less than or equal to a high school education (50%).

**Table 1:** Demographic characteristics of survey respondents

	Respondents (n = 944) n (%)
Age, mean (SD) 14–17 18–21 22–25	18.9 (2.8) 351 (37.2) 372 (39.4) 221 (23.4)
Gender	
Female	506 (53.6)
Male	359 (38.0)
Other gender identity	79 (8.4)
Race/Ethnicity	
Non-Hispanic Asian	128 (13.6)
Non-Hispanic Black	89 (9.4)
Hispanic	116 (12.3)
Mixed/Other	79 (8.4)
Non-Hispanic White	531 (56.3)
Participant education level	
Less than high school*	330 (35.0)
High school grad	146 (15.5)
Some college or tech school	278 (29.5)
Associate’s or tech grad	33 (3.5)
Bachelor’s degree or higher	157 (16.6)
School free or reduced lunch qualification	
Yes	339 (36.2)
No	597 (63.8)
Region	
Midwest	345 (36.6)
Northeast	142 (15.0)
South	261 (27.7)
West	196 (20.8)

*includes participants still in high school

After thematic review of the coded data, three key themes arose: (1) youth value personality, professionalism, accessibility, and the clinical training of healthcare providers; (2) youth most commonly cited gender concordance to be important, noting more comfort and open communication with similar providers; (3) respect and open communication make youth feel more comfortable and youth may switch doctors if they become uncomfortable (Table 2). Each of these key themes are discussed in greater detail below.

**Table 2:** Questions, themes, frequencies and example participant quotes

Theme	n (%)	Representative Quotes
**This week we want to know what you would look for when choosing a doctor. What characteristics are important to you, if any? Why? (n = 930)**		
**Personal factors**		
Kind, caring	291 (31.3)	“I want them to be friendly and someone I can easily approach”
Professional	201 (21.6)	“A doctor that focuses on patient care and not rushing through patients. A doctor who is focused and present” “Clarity, professionalism, cleanliness”
Listens, communicates	178 (19.1)	“Some one who LISTENS to me” “Someone who can communicate well with the patient”
Honest, trustworthy	119 (12.8)	“trustworthy, intelligent” “First of all safety is the most important. Someone you can trust to be honest with, because the honesty must go both ways in that kind of relationship”
Values	49 (5.3)	“mainly their stances on some public health issues like LGBTQ health”
**Logistical factors**		
Education, experience	279 (30.0)	“Experience, expertise, school they studied at”
Accessibility, affordability, location	181 (19.5)	“If it’s on my insurance, otherwise i can’t afford it” “Close to me, so basically the distance and probably what people say about them”
Reviews	173 (18.6)	“Good online reviews” “Their ratings to know I’m going to a good doctor that I can trust”
Services offered	57 (6.1)	“I personally look to see if they specialize in children or teen health”
**Demographic factors**		
Gender	84 (9.0)	“Must be the same gender as me” “I feel more comfortable with a doctor who is my own biological sex.”
Age	16 (1.7)	“A young doctor. So I can trust them” “Someone with good reviews and is middle age because they will be up to date with new medicine”
Race/Ethnicity	6 (0.6)	“Race because of how much it affects health outcomes”
**Is it important that you have similar characteristics to your doctor (age, gender, race/ethnicity, disability, background, personality, etc.)? If so, what characteristics? (n = 903)**		
Yes or depends	496 (54.9)	
*Gender*	339 (68.3)	“It’s helpful to have the same gender for me.”, “The only preference I really have is that the doctor is the same gender as me”
*Age*	74 (14.9)	“Depending on the person, yes. Ideally someone slightly younger because I associate that with being more tolerant or progressive. Also I prefer a womxn”
*Race or ethnicity*	73 (14.7)	“​​Yes, having a doctor similar to me in race usually helps put me at ease and relate better”
*Personality*	35 (7.1)	“Similar personality”, “yes, age and personality.”
*Background experiences*	26 (2.9)	“Similar gender and race, grew from similar socioeconomic background and understanding”
*Values or religious beliefs*	23 (2.5)	“I prefer seeing Jewish doctors. It’s just not the same.”, “Having similar values and personalities is important to me, as well as having the same gender.”
*LGBTQ + status*	22 (2.4)	“They must be gay”, “Yes sexual orientation”
*Disability status*	13 (2.6)	“I think it’s important to have as much in common with your doctor, particularly sex and disability, because those are harder to understand from someone that doesn’t have them”
*Language*	12 (1.3)	“Just gender and no language barrier”, “Yes that obviously helps, gender and race. Language too”
No	401 (44.4)	“Not really, I just want somebody I can trust and feel comfortable with”
**When would being similar to your doctor be important? Specifically, for what type of healthcare visits? (n = 852)**		
Always	74 (8.7)	“I’m not sure I feel like all health visits would be equally important”, “Any visit”
Sometimes	628 (73.7)	“General health visits, female health. Specialties would matter less”
Never	101 (11.9)	“Never”, “It wouldn’t matter”
Unsure	49 (5.8)	“I’m unsure”, “i have no idea”
**Types of visits**		
*Sexual health care*	405 (57.6)	“Sexual health visits primarily”, “Especially for women’s health or reproductive visits. Any visits that require a exam of my body”
*General care*	124 (17.6)	“general check ups! they will know how to make you feel comfortable and understand you”
*Mental health care*	71 (10.1)	“All kinds, but especially mental health”, “Maybe for psychological healthcare? Somebody who is able to empathize with one’s situation better.”
*Sensitive topics*	68 (9.7)	“I guess if I needed a personal one on one check up where they asked personal questions so I would feel less tense and more honest”
*Cultural health concerns*	50 (7.1)	“​​he could possibly understand the same kinds of struggles that i may face when it come to my health or culture”, “healthcare that your ethnicity might experience more often than others”
*Chronic health care*	27 (3.8)	“Chronic disease, cancer, genetic disease things that would require the support and explanation to my family”
*LGBTQ + health care*	20 (2.8)	“Ob-gyn. Queer identity-related visits.”, “For people who identify within the LGBT + community, having doctors of similar identities makes it easier to discuss specific needs”
**How would being similar to our doctor impact what you say or do during your visit? (n = 838)**		
More comfort	342 (40.8)	“I would feel more comfortable”
More open communication	307 (36.6)	“If your similar or you might be more open to sharing things with your doctor”, “I would find it easier to open up since I know they can relate better to how I feel or what health issues I have”
More understood	116 (13.8)	“it would make me feel understood and safe”
More trust	50 (6.0)	“If it were for say, mental illness I’d be more willing to take their input”, “I would probably trust them more”
No change	168 (20.0)	“I don’t think it would at all”, “It wouldn’t”
It depends	59 (7.0)	“It rlly depends on the issue it’s kind hard to tell rlly”
I don’t know	41 (4.9)	“Idk”
**If you were in a situation where you felt uncomfortable with your doctor, what could they do to make you feel more comfortable? (n = 821)**		
**Doctor behavior change**		
Be nice and respectful	167 (20.3)	“Treat me with respect”, “Make themselves approachable and kind”
Communicate more	114 (13.9)	“Take a second and just talk, get to know each other a bit. Serves 2 purposes: a break from the uncomfortable situation, and a chance to feel more comfortable with the doctor”
Address the issue	108 (13.2)	“Talk to them about it and just be honest”, “address the issue”
Provide reassurance	103 (12.5)	“Just be patient and keep ensuring me that nothing I say will leave the room”, “Reassurance of their ideas/ diagnoses, that they’re confident that they’re able to help me with my health issues”
Listen and empathize	84 (10.2)	“They could try to relate to me better as a person rather than a patient”, “Listen to all of my problems and not just write them off as nothing to worry about”
Use humor or build rapport	68 (8.3)	“Maybe make a joke or something along those lines”, “Tell a bit about themselves so I know where they’re coming from”
**Change in setting**		
Switch doctors	107 (13.0)	“If I was uncomfortable with my doctor I just ask for another one”
Request chaperone	59 (7.2)	“Get a female doctor or nurse to come in as well during my visit”, “Let me bring someone else into the room”
**I don’t know**	150 (18.3)	“I’m not sure”, “I don’t know”

### Youth value personality, professionalism, and accessibility in addition to clinical training of healthcare providers

While 30% (279/930) of youth respondents noted education or intelligence as an important factor in choosing their doctor, a variety of other factors also emerged. Youth most commonly reported that “kindness, easygoing, friendly, and non judgemental” or other personality traits (31%; 291/930) were important in choosing their doctor. Others noted factors like “Cordiality, timeliness, and professionalism” (22%; 201/930), accessibility related to being “covered by my insurance…located near me, has availability to schedule appointments” (19%; 181/930), and “easy to talk to and explains information in a way that’s easy for me to understand” (19% (178/930). Some youth even noted they would rely on word of mouth or “good ratings” and reviews to help them choose a doctor (19%; 173/930).

### Youth most commonly cited gender concordance to be important, noting more comfort and open communication with similar providers

When prompted about the importance of patient-physician similarity, almost half of youth respondents (44%; 401/903) noted that similarity with their physician was not important to them or that personality and relationship were more important.

No, I don’t care about my physician’s physical/social traits aside from personality. They need to be kind and considerate, but aside from that, I don’t care if they’re male, female, old, young, disabled, normally abled, poor, rich, white, black, brown, or even green! As long as they are a good person who cares, understands, and offers scientific treatment, I am happy with them.

About one third of respondents (37% 337/903) noted similarities were important while others said it depended on the situation or that it mattered for some characteristics but not others (18%; 159/903). Among this group that reported a preference for patient-physician concordance (55%; 496/903), most (68%; 339/496) noted that “It’s helpful to have the same gender for me.” Other factors noted by youth included age (“I prefer younger doctors just because it makes me more comfortable.”; 15%), race or ethnicity (“yes, my pediatrician is a black woman which is important for my because i am those things as well”; 15%), and personality (“A similar personality would be nice as well as similar beliefs”; 7%). Less commonly noted characteristics included values or religious beliefs, background (socioeconomic, life experiences, etc.), LGBTQ + status, and disability status.

Respondents identifying as female were more likely to report concordance as important overall compared to non-female respondents (female: 46% vs. male: 26% and other identity: 38%; *p* < 0.0001) and more likely to note a desire for gender concordance specifically (female: 81% vs. male: 43% and other identity: 55%; *p* < 0.0001). Among respondents noting any preference for concordance, non-white and low-income participants were more likely to note a preference for racial concordance (29% vs. 4%; *p* < 0.0001 and 21% vs. 12%; *p* = 0.0089 respectively) and less likely to note a preference for gender concordance (62% vs. 73%%; *p* = 0.0103 and 52% vs. 75%; *p* < 0.0001 respectively) compared to their counterparts. Within the full cohort 8.1% of respondents noted a preference for racial concordance (15.9% of non-white and 2.2% of white individuals, *p* < 0.0001). Black respondents were most likely to report preference for racial concordance (25%) compared to individuals with Asian (15%), Hispanic (14%), white (2%), and other (8%) racial identities (*p* < 0.0001).

When asked when being similar to their doctor would be important, most respondents (74%; 628/852) again noted that similarity would matter only in specific instances and a few (9%; 74/852) noted it would always be important. Sexual health related care was the most commonly noted scenario in which similarity would be important (58%; 405/702). Within this group, most (76%; 308) specified the importance of similarity for female health issues, “Gynecologist, anything that is gender specific,” with fewer individuals (10%; 40) reporting on male specific issues like, “Male doctor for male things.”

Participants identifying as male were least likely to report that being similar was always or sometimes important (male: 70% vs. female: 90% and other identity: 86%; *p* < 0.0001) with females most likely to report importance for sexual health visits (female: 68% vs. male: 38% and other identity: 59%; *p* < 0.0001) compared to other genders. Non-white and low-income individuals were more likely to report similarity would be important for cultural or language related concerns (12% vs. 4%; *p* < 0.0001 and 13% vs. 4%; *p* < 0.0001 respectively) compared to their peers and less likely for sexual health visits (51% vs. 63%; *p* = 0.0024 and 51% vs. 61%; *p* = 0.0124 respectively).

In regard to the impact similarity would have on youth during a visit, respondents most commonly noted it would “Make me feel more comfortable disclosing personal information” (41%; 342/838) or “It would let me be more open and honest” (37%; 307/838). They also noted they “might be more honest with someone who understands my culture and is not judging” (14%; 116/838). Many youths however noted it would have no impact (20%; 168/838) or they were unsure how it would impact what they said or did during a visit (5%; 41/838).

Non-cisgendered respondents were most likely to report that similarities would help youth feel more understood (other identity: 26% vs. female: 14% and male: 10%; *p* = 0.0015) and create more open communication (other identity: 55% vs. female: 37% and male: 30%; *p* = 0.0003) while females were most likely to report being similar would help them to feel comfortable (female: 50% vs. male: 27% and other identity: 28%; *p* < 0.0001). Non-white participants were more likely to report similarities would help them feel more understood (17% vs. 11%; *p* = 0.0035) compared to white participants.

### Respect and open communication make youth feel more comfortable and youth may switch doctors if they become uncomfortable

When youth were asked about what would make them feel more comfortable if they felt uncomfortable with their doctor, youth noted both situational and provider specific ideas. Youth most commonly (20%; 167/821) noted that their doctor should just “treat me with respect” or “just have more open conversation overall” (14%; 114/821) in these instances. Respondents also suggested they “address the discomfort and talk through it” (13%; 108/821), “be reassuring” (13%; 103/821), and “Be empathetic and truly listen” to their concerns (10%; 84/821).

From a situational perspective, a number of respondents (7%; 59/821) noted that their doctor could bring a chaperone into the room to help them feel more comfortable: “have another person in the room to help mediate” or “Possibly have another female present.” Another group of respondents (13%; 108/821) however noted that if they were made uncomfortable by a doctor there was nothing to be done and they would simply switch providers or want their provider to provide a referral to another provider - “I would switch right away.”

## Discussion

Youth respondents in our study valued providers’ personality and empathy when choosing their provider. Demographic characteristics such as age, gender, and race/ethnicity were less commonly noted to be important. When reported, gender concordance was most often noted by self-reported female respondents and in the context of sexual health visits. Non-white and low-income respondents were more likely to prefer racial concordance than their peers, though desire for racial concordance was low for both groups. Participants also noted that uncomfortable situations could in part be mitigated by respect and open communication, though some youth preferred to simply switch providers.

Across all respondents, concordance was most important in relation to gender and, relatedly, in the provision of sexual health related care. These results are similar to prior research in adults that has suggested gender concordance to be particularly important to patients and tied to perceived quality of care [28, 29]. Preference for racial concordance in our sample was primarily reported by non-white participants. This may be expected since the majority of physicians in the United States identify as white, making racial concordance inherently more likely for white youth [30, 31]. Additionally, non-white participants were more likely than white respondents to prefer providers that had similar cultural backgrounds or language fluency, which is similar to past studies that demonstrate that minority or non-English speaking patients prefer racial and language concordance [28, 32]. However, overall, the percentages of non-white youth and youth overall reporting that racial concordance was important was relatively low (16% and 8% respectively), with some youth reporting explicitly that it was not important. Evidence from past studies is mixed on the impact of concordance on healthcare [20, 33, 34], with the exception of language concordance [35, 36]. Healthcare providers may better engage and support youth as they develop more independence in their own healthcare if youth are provided options and are guided in selecting a provider that would be a good fit. Online reviews or ratings, which are critical to many consumers [37], may be of particular interest to youth in selecting a provider and these avenues were already noted by respondents in our study. Similarly, education regarding how to change their provider may empower youth to “shop” for a provider that best fits their needs by researching online or via their social network, rather than avoiding care if/when they have uncomfortable experiences with a provider.

Many youths also mentioned the importance of respect, compassion, and communication within patient-provider relationships. These perspectives are similar to previous research that highlighted the importance of providers being “youth-friendly” [38]. This research, as well as guidelines for youth care, note the importance of ensuring good communication so youth feel heard and understand their care plan. Youth-friendly care also includes creating an environment that is friendly, respectful, non-patronizing, non-judgmental, honest, and unbiased among others [38–43]. These insights emphasize educating and preparing providers and healthcare administrators to implement these tangible and intangible factors. Programs such as the Adolescent-Centered Environment Assessment Process (ACE-AP) are designed to assess and improve the adolescent-friendliness of clinics and have been found to improve the quality and satisfaction of care provided to youth [44]. Inclusion of training on concepts such as cultural humility [45] and structural competency [46] may additionally enable providers to care for youth in a respectful and supportive way. The Association of American Medical Colleges has also developed a professional readiness exam (PREview) for pre-medical students that assesses cultural competence, social skills, and listening skills. These new assessments demonstrate the value that medical schools are beginning to place on these skills [47, 48].

Similarly, if concerns were to arise where youth felt uncomfortable with a provider, respondents in our sample favored providers being “nice,” “respectful,” and addressing the issue directly to ease their discomfort. Though directly addressing uncomfortable situations may be difficult for providers, youth have previously reported higher satisfaction when sensitive topics were addressed at their visit [49] as well as appreciation of direct communication [38]. However, as mentioned by participants in this study, some youth may simply need to change providers to improve their care experience [38]. In these situations, it may be important for providers and clinic staff to offer alternative options so youth understand that they can ask for a new provider who they may feel more comfortable with or connected to. Opportunities to connect with adolescent and young adult specialists, who are specifically trained to support this population, during this period of transition may also be an important option [50]. Efforts to accommodate patients’ preferences for patient-physician concordance should be undertaken with care, so as to not exacerbate the minority tax [51] on physicians from historically marginalized backgrounds in medicine. This can potentially be accomplished by diversifying the physician workforce and by training all healthcare providers to better deliver care across differences.

### Limitations

There are several limitations of this study. The MyVoice sample is not nationally representative and therefore responses may not be generalizable. The questions asked were also hypothetical and may not match their behaviors as we did not ask youth about their actual interactions in healthcare to protect their confidentiality. Some respondents were also under age 18 and may have limited experiences with managing their healthcare independently. As in all surveys, it is also possible that youth’s responses are subject to desirability bias, though our findings included many responses that are critical and counter to current norms.

## Conclusions

In conclusion, our findings suggest that personality and empathy are important provider characteristics valued by youth. Youth in our sample that identified as female were more likely to prefer gender concordant providers, particularly for sexual health related issues, and non-white respondents were more likely to prefer racial concordance compared to peers. Programs that assess and strengthen professional and interpersonal skills such as listening and cultural humility may improve engagement and satisfaction among adolescent and young adult patients, particularly non-white and female patients. Addressing uncomfortable interactions directly or switching providers are approaches youth in our sample endorsed.

## Data Availability

The datasets used and/or analyzed during the current study are available from the corresponding author on reasonable request.
